# Identification of Diagnostic Biomarkers in Systemic Lupus Erythematosus Based on Bioinformatics Analysis and Machine Learning

**DOI:** 10.3389/fgene.2022.865559

**Published:** 2022-04-14

**Authors:** Zhihang Jiang, Mengting Shao, Xinzhu Dai, Zhixin Pan, Dongmei Liu

**Affiliations:** ^1^ Department of Rheumatology and Immunology, Shengjing Hospital, China Medical University, Shenyang, China; ^2^ Computational Systems Biology Laboratory, Department of Bioinformatics, Shantou University Medical College (SUMC), Shantou, China

**Keywords:** systemic lupus erythematosus, machine learning, integrated bioinformatics, diagnostic biomarkers, immune infiltration

## Abstract

Systemic lupus erythematosus (SLE) is a complex autoimmune disease that affects several organs and causes variable clinical symptoms. Exploring new insights on genetic factors may help reveal SLE etiology and improve the survival of SLE patients. The current study is designed to identify key genes involved in SLE and develop potential diagnostic biomarkers for SLE in clinical practice. Expression data of all genes of SLE and control samples in GSE65391 and GSE72509 datasets were downloaded from the Gene Expression Omnibus (GEO) database. A total of 11 accurate differentially expressed genes (DEGs) were identified by the “limma” and “RobustRankAggreg” R package. All these genes were functionally associated with several immune-related biological processes and a single KEGG (Kyoto Encyclopedia of Genes and Genome) pathway of necroptosis. The PPI analysis showed that IFI44, IFI44L, EIF2AK2, IFIT3, IFITM3, ZBP1, TRIM22, PRIC285, XAF1, and PARP9 could interact with each other. In addition, the expression patterns of these DEGs were found to be consistent in GSE39088. Moreover, Receiver operating characteristic (ROC) curves analysis indicated that all these DEGs could serve as potential diagnostic biomarkers according to the area under the ROC curve (AUC) values. Furthermore, we constructed the transcription factor (TF)-diagnostic biomarker-microRNA (miRNA) network composed of 278 nodes and 405 edges, and a drug-diagnostic biomarker network consisting of 218 nodes and 459 edges. To investigate the relationship between diagnostic biomarkers and the immune system, we evaluated the immune infiltration landscape of SLE and control samples from GSE6539. Finally, using a variety of machine learning methods, IFI44 was determined to be the optimal diagnostic biomarker of SLE and then verified by quantitative real-time PCR (qRT-PCR) in an independent cohort. Our findings may benefit the diagnosis of patients with SLE and guide in developing novel targeted therapy in treating SLE patients.

## Introduction

Systemic lupus erythematosus (SLE) is a chronic autoimmune disease mediated by autoimmune reactions and is characterized by autoimmune inflammation (Bakshi et al., 2018). The serum of SLE patients contains a variety of autoantibodies represented by antinuclear antibodies, which combined with the corresponding autoantigens in the body to form immune complexes and then deposited in the glomerulus, liver, joints, skin, and other parts, causing multiple symptoms and manifestations (Ahlin et al., 2012). Currently, the pathogenesis and etiology of SLE have not been fully elucidated, and biomarkers available in clinical practice are still limited, mainly including anti-dsDNA antibodies, complement molecules, and white blood cell counts (Piga and Arnaud, 2021). Because of the diversity of symptoms, it can sometimes be difficult to distinguish SLE from other diseases with similar symptoms, such as rheumatoid arthritis (RA) and myositis. However, early diagnosis and timely intervention can help reduce SLE recurrence and hospitalization rates and the accumulation of chronic organ damage (Mak et al., 2013; Piga and Arnaud, 2021). Therefore, the identification of reliable biomarkers and revealing the underlying molecular mechanisms are essential for better diagnosis and effective treatment of SLE.

In recent years, numerous biomarkers in the different processes of autoimmune diseases have been identified through comprehensive bioinformatics analyses, including SLE (Zhao et al., 2021), RA (Cheng et al., 2021) and ulcerative Colitis (Chen et al., 2020), laying the foundations for exploring the potential molecular mechanisms in autoimmune diseases. Meanwhile, with the rapid development of artificial intelligence (AI), machine learning algorithm, as an important branch, has been widely used in the diagnostic classification and prognostic prediction of diseases. For example, machine learning methods were used to identify key prognostic molecules in esophageal squamous cell carcinoma (Li et al., 2021). Potential diagnostic biomarkers of acute myocardial infarction were identified by the least absolute shrinkage and selection operator (LASSO) regression model and support vector machine recursive feature elimination (SVM-RFE) (Zhao et al., 2020). Machine learning algorithms are generally divided into weak classifier algorithms and strong classifier algorithms. For example, logistic regression (LR), support vector machine (SVM), and artificial neural network (ANN) are weak classifier algorithms, and random forests (RF) and extreme gradient enhancement (XGBoost) are strong classifier algorithms. A strong classifier can be composed of more than one weak classifier. As proposed by Stafford et al., depending on the high classification performance of clinical and genomic data, RF and SVM were most frequently utilized in the diagnosis of autoimmune diseases (Stafford et al., 2020).

Therefore, based on bioinformatic analyses and machine learning algorithms, the present study was aimed to identify potential diagnostic biomarkers in SLE and construct the molecular regulatory networks related to diagnostic biomarkers, laying a foundation for in-depth exploration of molecular mechanisms of SLE.

## Materials and Methods

### Data Sources

The expression profiles of 924 SLE and 48 control samples in GSE65391, 99 SLE and 18 control samples in GSE72509, and 78 SLE and 46 control samples in GSE39088 were downloaded from the GEO database (https://www.ncbi.nlm.nih.gov/geo/). GSE65391 and GSE39088 were used to identify potential diagnostic biomarkers in SLE, while GSE39088 was used to test the reliability of these diagnostic biomarkers.

### Screening and Functional Analysis of DEGs

DEGs between SLE and control samples in GSE65391 and GSE39088 datasets were identified by the “limma” R package with the threshold |log_2_FC| >1 and *p*-value < 0.05. The general views of DEGs were shown as volcano plots. The expressions of DEGs were visualized in the heatmap. The volcano plots and heatmaps were generated by the “ggplot2” R package. “RobustRankAggreg” R package was performed to screen accurate DEGs from GSE65391 and GSE39088 (Zhou et al., 2021). Then the functions of screened DEGs were analyzed by the “clusterProfiler” R package. *p*-value < 0.05 was considered as significantly enriched.

### Identification of Diagnostical Biomarkers and Prediction of Regulators and Drugs

The screened DEGs were submitted into the STRING database (https://string-db.org), and then a PPI network was constructed by setting the confidence as 0.4. The correlations among DEGs in the PPI network were evaluated by the Pearson method and visualized in the heatmap. Then the expressions of correlated DEGs (Cor >0.7) were tested in GSE39088. Thereafter, the performance of these DEGs in distinguishing SLE and control samples were evaluated by ROC curves analyses, and DEGs with AUC greater than 0.7 were identified as diagnostic biomarkers of SLE. Furthermore, the reliability of these biomarkers was tested in GSE39088. The miRWalk database (http://mirwalk.umm.uni-heidelberg.de/) was used to predict the miRNAs targeting these diagnostic biomarkers. The ChEA3 database (https://amp.pharm.mssm.edu/chea3/) was used to predict the TFs targeting diagnostic biomarkers. Then the miRNA-diagnostic biomarker and TF-diagnostic biomarker pairs were integrated into a miRNA-diagnostic biomarker-TF regulatory network and visualized by Cytoscape software. The CTD database (http://ctdbase.org/) was used to search for drugs targeting these diagnostic biomarkers, and the PubChem database (http://www.pubchem.ncbi.nlm.gov) was used to display the chemical structures of several drugs, and a drug-gene network was constructed and visualized.

### Evaluation of Immune Cell Infiltration

The immune infiltrations of 28 types of immune cells in SLE and control samples from GSE65391 were evaluated by the ssGSEA method (Ye et al., 2019). Differentially infiltrated immune cells between SLE and control samples were identified by the Wilcoxon test using *p*-value < 0.05 as the cutoff. Furthermore, the correlations between differentially infiltrated immune cells and diagnostic biomarkers were calculated by the Spearman method and shown in the heatmap.

### Machine Learning

Machine learning methods, including LR, RF, XGBoost, SVM, and ANN (Li et al., 2021), were performed by the “glmnet”, “randomForest”, “xgboost”, “e1071”, and “neuralnet” R packages to develop classifiers for diagnostic classification, respectively. For each machine learning algorithm, 1,023 models representing all combinations of 10 identified biomarkers were established, and AUCs of the models were calculated. Among all classifiers, the top 100 models with the highest AUC values were selected, and the occurrence frequencies of each diagnostic biomarker were counted. The top five diagnostic biomarkers with the highest occurrence frequency in every classifier were extracted and overlapped to identify the optimal diagnostic biomarkers.

### Collection of Clinical Characteristics

A total of 26 SLE patients and 20 sex- and age-matched healthy controls were recruited from Shengjing Hospital of China Medical University. The clinical characteristics of subjects, such as age, sex, course of the disease, and clinical and laboratory indices, were obtained from electronic medical records. The diagnosis of SLE was followed according to the European League Against Rheumatism (EULAR)/American College of Rheumatology (ACR) 2019 criteria and the SLE disease activity was assessed according to the systemic lupus erythematosus disease activity index 2000 (SLEDAI-2K) (Gladman et al., 2002). Patients with malignant tumors, pathogen infection, and other autoimmune diseases, such as RA and systemic sclerosis, were excluded. The research protocol was approved by the Medical Ethics Committees of the Shengjing Hospital of China Medical University. All experiments were conducted by the principles and regulations formulated by the ethics committee.

### RNA Extraction and qRT-PCR

Blood samples from each subject were collected in ethylenediaminetetraacetic (EDTA) tubes. Peripheral blood mononuclear cells (PBMCs) were obtained by density gradient centrifugation (Solarbio Life Sciences, Beijing, China). Total cellular RNA was extracted from PBMCs using RNA Extraction Kit (Omega, Guangzhou, China). 200 ng RNA per sample was submitted for reverse transcription using Evo M-MLV RT Kit (Accurate Biotechnology, Changsha, China) following the manufacturer’s instructions. The qPCR cycle was conducted using SYBR Green Premix Pro Taq HS qPCR Kit (Accurate Biotechnology, Changsha, China) on Light Cycler 480 real-time PCR instrument (Roche, Basel, Switzerland). The 2^−ΔΔCt^ method was used to calculate the relative expression of mRNA. β-actin was used as the internal control for normalization. The gene-specific primers are available in Supplementary Table S1.

### Statistical Analysis

All the statistical analyses were performed with R software (version 4.1.0). The student’s t-test was performed to compare gene expressions between different groups. ROC curve analysis was used to evaluate the performance of biomarkers for diagnosing SLE. *p* < 0.05 was considered statistically significant.

## Results

### DEGs Involved in SLE

**FIGURE1:**
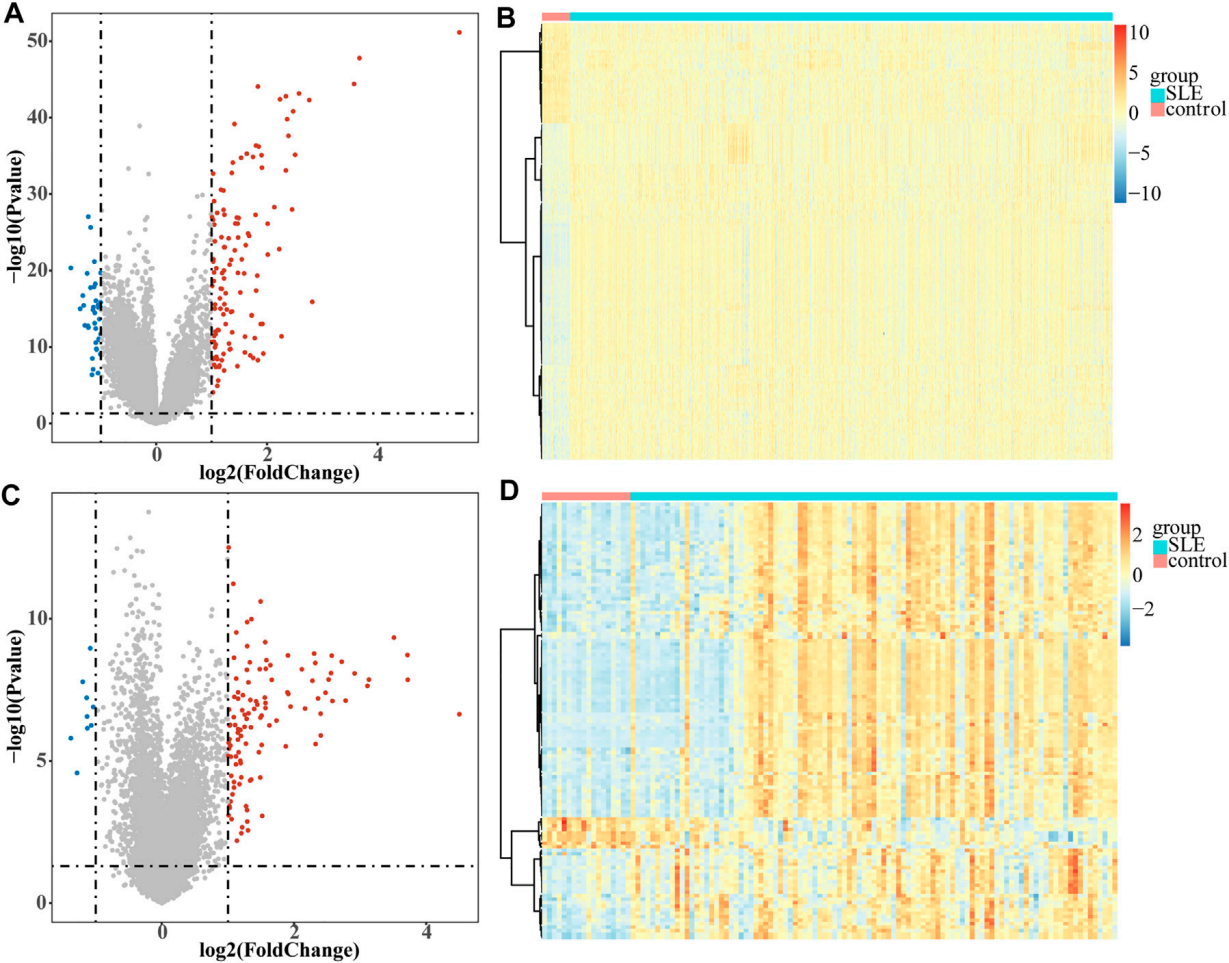
Volcano plots and heatmaps of DEGs. Each colored dot represents a DEG (|log2FC| >1 and *p*-value < 0.05). The blue dots represent the downregulated genes and the red dots represent the up-regulated genes. **(A)** Volcano map of the 161 EDGs identified in GSE65391. **(B)** Heatmap of the 161 EDGs identified in GSE65391. **(C)** Volcano map of the 125 EDGs identified in GSE72509. **(D)** Heatmap of the 125 EDGs identified in GSE72509.

**FIGURE 2:**
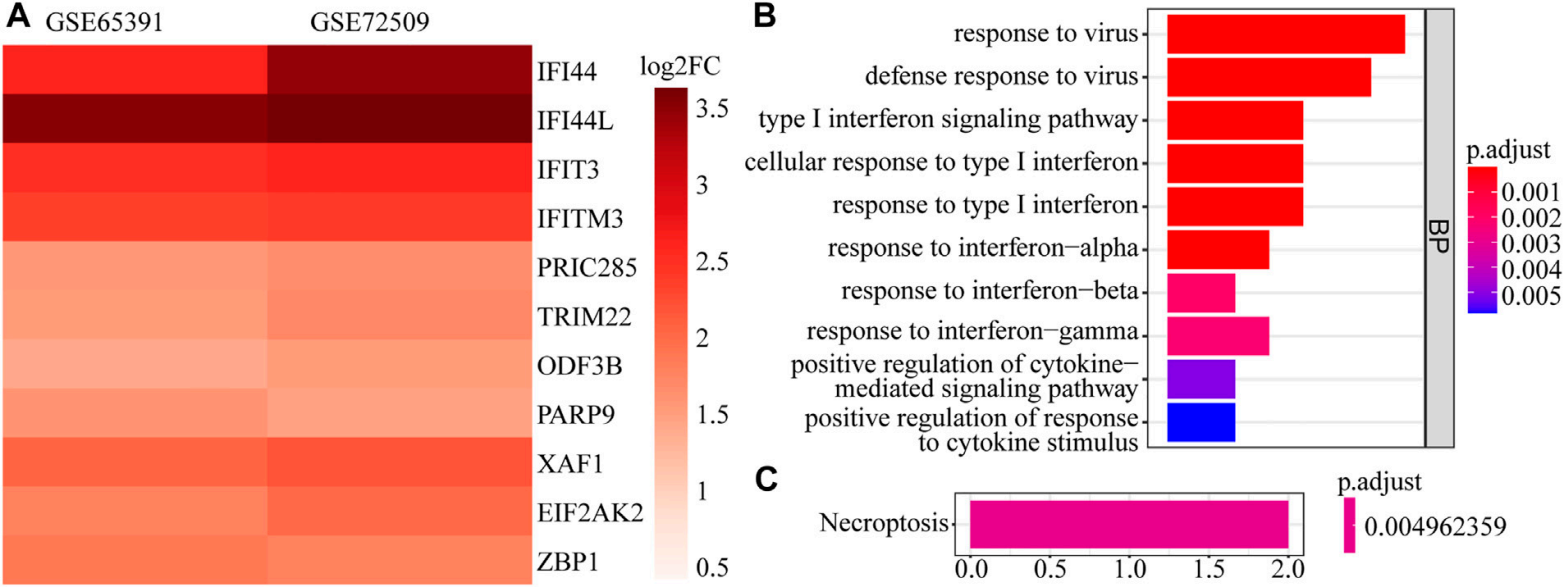
The expression heatmap and enrichment analysis of accurate DEGs. **(A)** The expression heatmap of 11 accurate DEGs in GSE65391 and GSE72509. **(B)** The top 10 enriched GO terms. **(C)** The enriched pathway.

A total of 161 DEGs, including 124 up-regulated and 37 down-regulated genes in SLE samples relative to control samples, were identified in GSE65391 (Supplementary Table S2; Figure 1A), and the expression levels were shown as a heatmap (Figure 1B). Meantime, a total of 125 DEGs, including 116 up-regulated and nine down-regulated genes in SLE, were identified in GSE72509 (Supplementary Table S3; Figure 1C), and the expression levels were also shown as a heatmap (Figure 1D). By RobustRankAggreg method, IFI44, IFI44L, EIF2AK2, IFIT3, IFITM3, ZBP1, TRIM22, PRIC285, XAF1, PARP9, and ODF3B were screened as accurate DEGs (Supplementary Table S4). All the expressions of them were up-regulated in SLE samples (Figure 2A). The top 10 biological processes into which these DEGs were significantly enriched were associated with immunity, such as response to virus, type Ⅰ interferon signaling pathway, cellular response to type Ⅰ interferon, and positive regulation of cytokine-mediated signaling pathway (Figure 2B). In addition, these DEGs were markedly associated with a KEGG pathway of necroptosis (Figure 2C).

### Ten Diagnostic Biomarkers Identified in SLE

**FIGURE 3:**
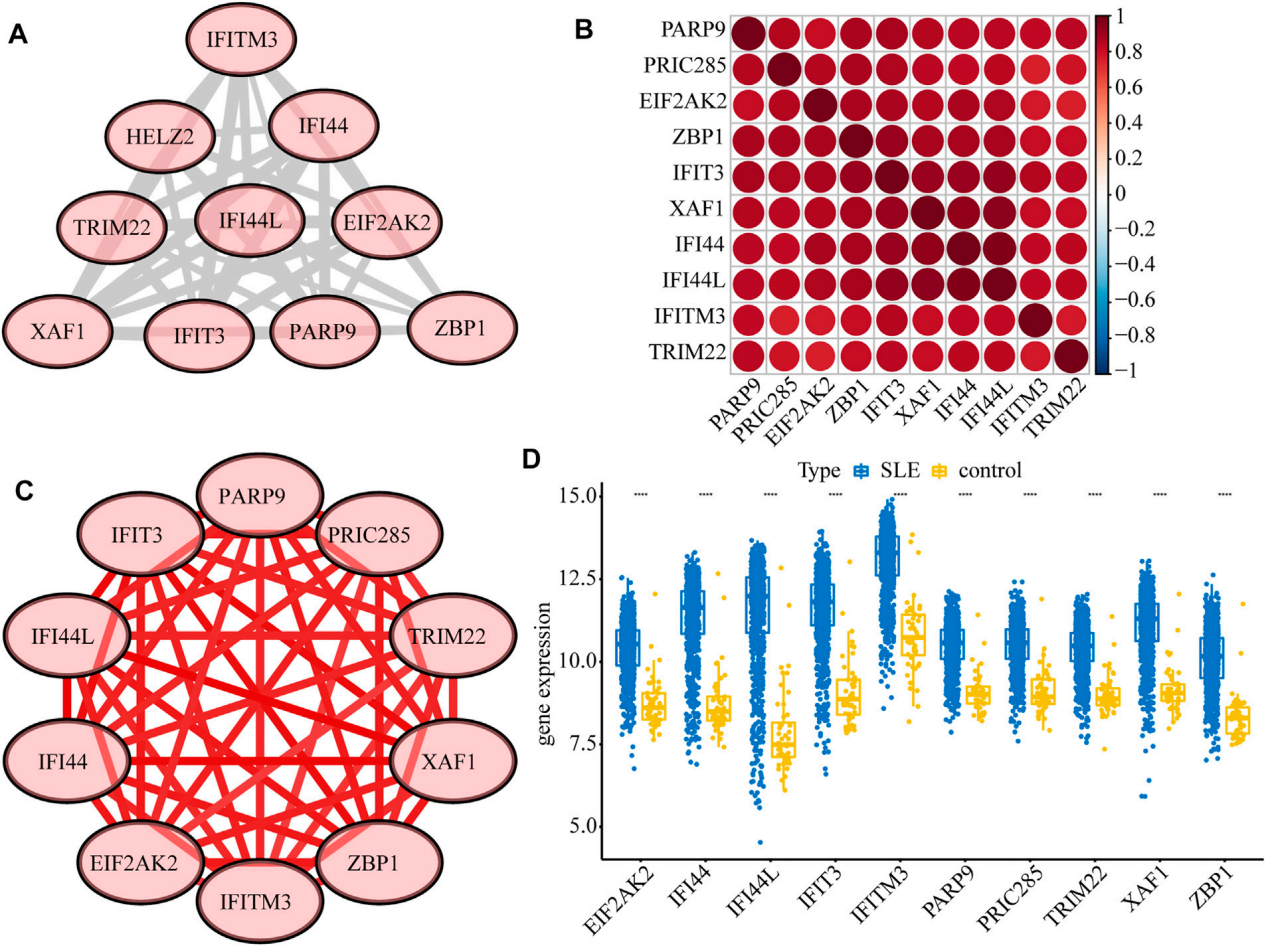
Interactions among the ten diagnostic biomarkers. **(A)** The PPI network of the ten biomarkers. **(B)** The correlation heatmap of the ten biomarkers. **(C)** The correlation network of the ten biomarkers. **(D)** The boxplot of the expression levels of ten biomarkers validated in GSE39088.

**FIGURE4:**
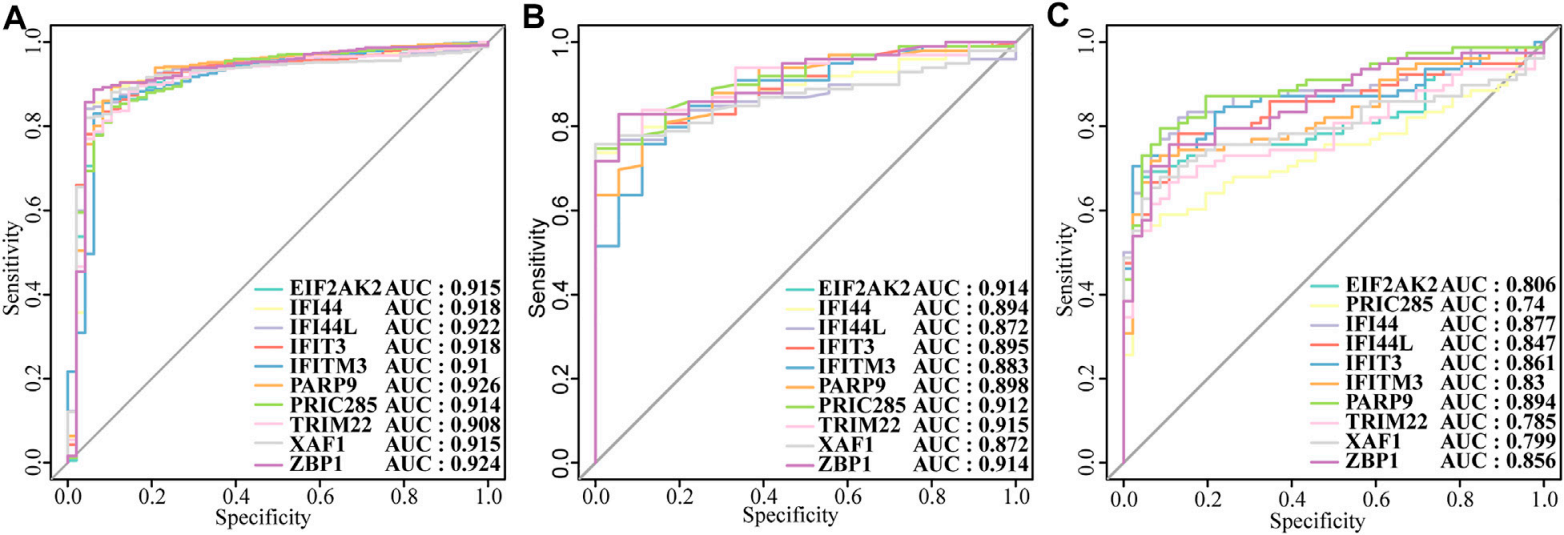
The diagnostic performance of the ten biomarkers. **(A)**Training set: GSE65391. **(B)** Validation set: GSE72509. **(C)** Test set: GSE39088.

Next, we constructed a PPI network of the DEGs, including IFI44, IFI44L, EIF2AK2, IFIT3, IFITM3, ZBP1, TRIM22, PRIC285 (also named as HELZ2), XAF1, and PARP9 (Figure 3A). These 10 genes had strong positive correlations with each other (Cor >0.7, Figure 3B), so we constructed a correlation network according to their correlations (Supplementary Table S5; Figure 3C). Moreover, the expression patterns of these 10 genes were validated in the GSE39088 dataset and were all up-regulated in SLE samples (Figure 3D). To identify their performance in distinguishing SLE and control samples, we plotted ROC curves in GSE65391 GSE72509 and GSE39088 datasets. The AUCs of the 10 genes were higher than 0.9 in GSE65391 (Figure 4A), higher than 0.85 in GSE72509 (Figure 4B), and higher than 0.7 in GSE39088, indicating that these genes had high accuracy and reliability in distinguishing between SLE and control samples. Thus, these 10 genes were identified as diagnostic biomarkers in SLE.

### Construction of the TF-Diagnostic Biomarker-miRNA Network

**FIGURE 5:**
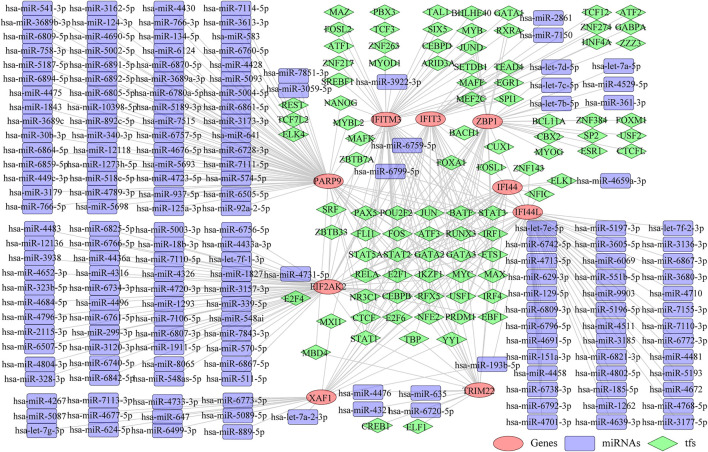
TF-biomarker-miRNA regulatory network. The orange ellipses represent the biomarkers, the purple round rectangles represent the miRNAs and the green diamonds represent the TFs.

**FIGURE 6:**
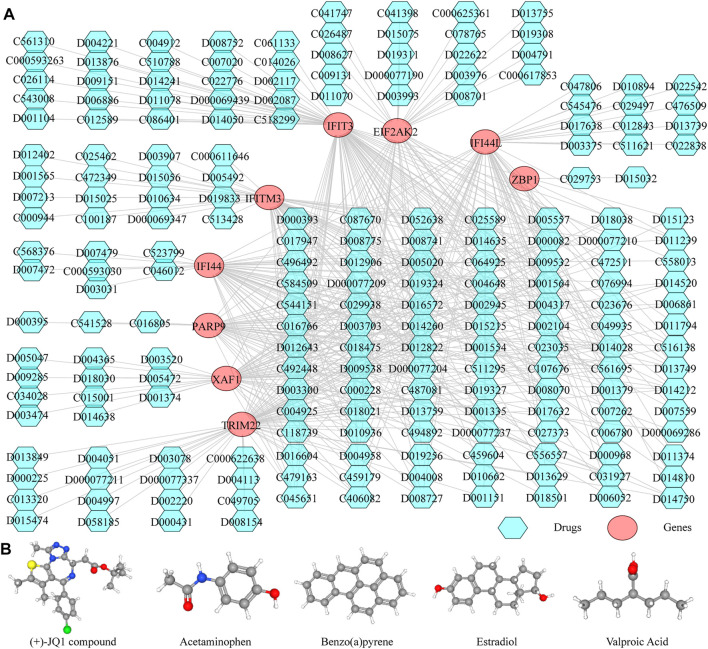
Predicting the drugs targeting the diagnostic biomarkers. **(A)** Drug-gene network. **(B)** Chemical structures of few of the drugs, from left to right, are (+)-JQ1 compound, acetaminophen, Benzo**(A)**pyrene, Estradiol and Valproic Acid.

Thereafter, we investigated the miRNAs and TFs that regulate the expression of the identified biomarkers. By miRWalk database, 176 miRNAs targeting IFI44, IFI44L, EIF2AK2, IFIT3, IFITM3, ZBP1, TRIM22, XAF1, and PARP9 were predicted, and a miRNA-biomarker network composed of 185 nodes and 179 edges was constructed (Supplementary Figure S1). In addition, 93 TFs binding with and regulating the expressions of IFI44, IFI44L, EIF2AK2, IFIT3, IFITM3, ZBP1, TRIM22, XAF1, and PARP9 were obtained from the ChEA3 database, and a TF-biomarker network composed of 102 nodes and 226 edges was constructed (Supplementary Figure S2). After integration, a TF-biomarker-miRNA regulatory network was visualized by Cytoscape, including 278 nodes and 405 edges (Figure 5). Furthermore, we also predicted the drugs targeting the biomarkers by CTD (Supplementary Table S6), extracted the drug-biomarker relation pairs, and constructed a drug-gene network composed of 218 nodes and 459 edges, including nine biomarkers and 209 drugs (Figure 6A). The chemical structures of several drugs, including (+)-JQ1 compound, acetaminophen, Benzo(a) pyrene, Estradiol, and Valproic Acid are displayed in Figure 6B.

### Immune Cell Infiltration Results

**FIGURE 7:**
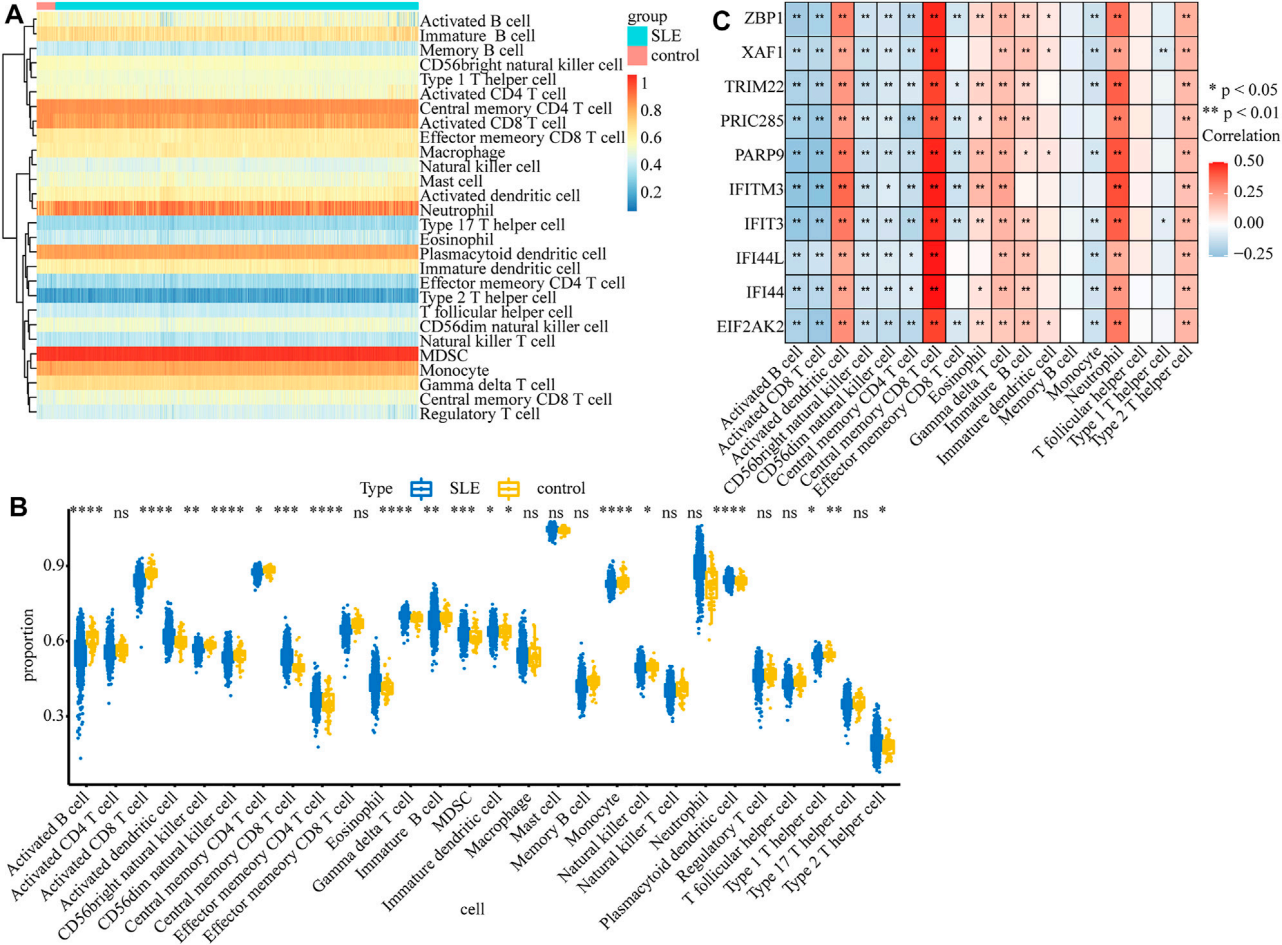
The relationship between diagnostic biomarkers and immune cell infiltration. **(A)** The heatmap of the infiltration proportion of 28 types of immune cells. **(B)**The boxplot of the infiltration proportion of 28 types of immune cells in SLE and control samples. **(C)** The heatmap of the correlations between diagnostic biomarkers and immune cells.

Considering that SLE is an autoimmune disease and immune cells play important roles in affecting disease progression, we analyzed and compared the immune infiltration in SLE and control samples. The infiltration of 28 types of immune cells in each sample was calculated by the ssGSEA algorithm (Supplementary Table S7) and the results are displayed in the heatmap (Figure 7A). We found that the infiltrations of most immune cells were significantly different between SLE and control samples (Figure 7B). Moreover, we found that the expression of biomarker genes was positively correlated with activated dendritic cells, central memory CD8 T cells, gamma delta T cells, neutrophils, and type 2 T helper cells, but negatively correlated with activated B cells, activated CD8 T cells, CD56bright natural killer cells, CD56dim natural killer cells and central memory CD4 T cells (Figure 7C), indicating that these biomarkers may regulate SLE via interplay with the immune environment.

### Identification of Optimal Biomarkers in SLE

**FIGURE 8:**
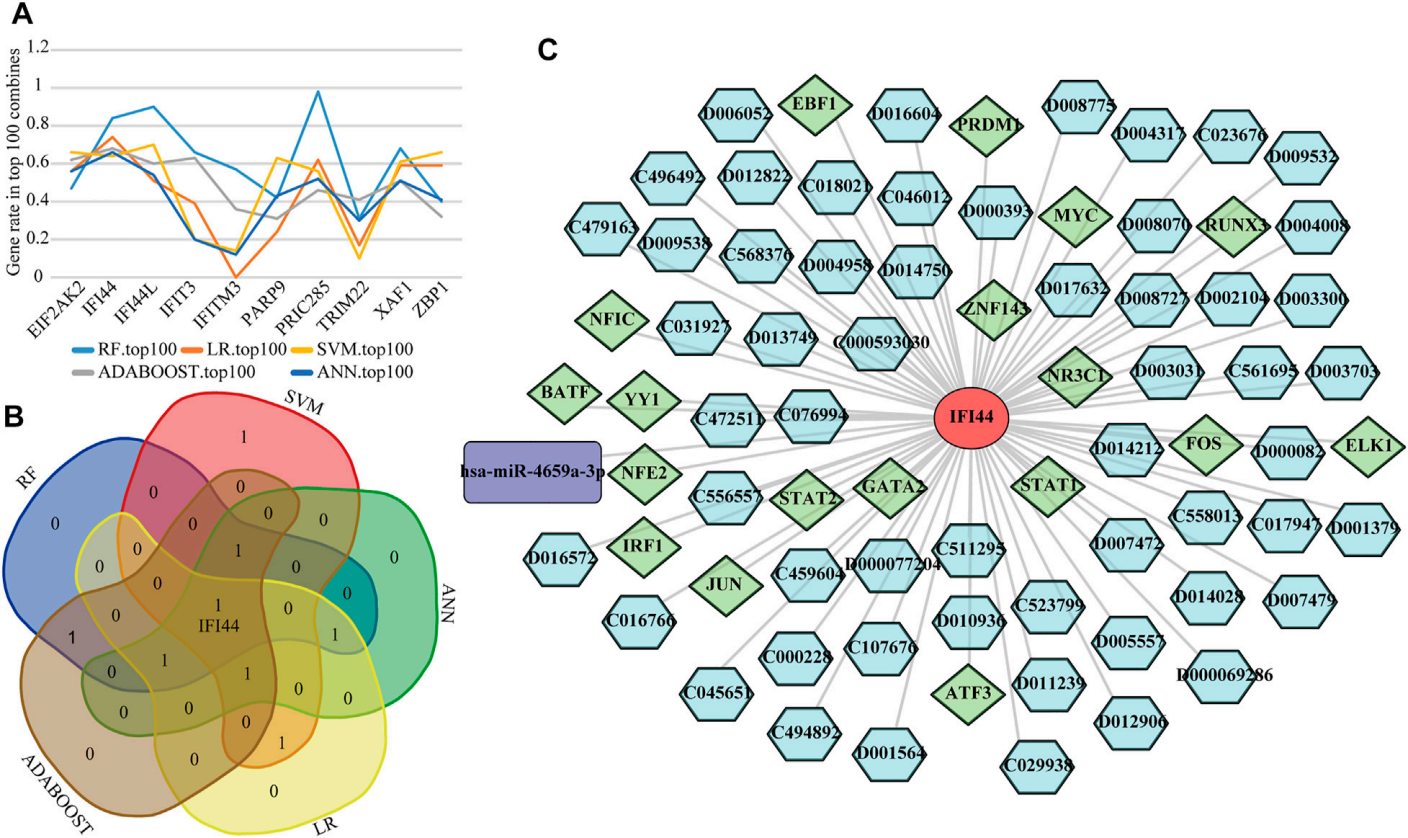
Identification of the optimal diagnostic biomarkers. **(A)** The occurrence frequencies of ten diagnostic biomarkers in top 100 models. **(B)** Venn diagram of top five important biomarkers in each machine learning analysis. **(C)** The IFI44 related network.

To detect the optimal SLE biomarkers, we performed machine learning analyses, in which the importance of these 10 biomarkers was weighted by their occurrence frequencies in the top 100 models (Figure 8A). The top five important biomarkers in each machine learning method were shown in Table 1, and the only intersecting biomarker was IFI44 (Figure 8B), indicating that IFI44 was the optimal SLE biomarker. Then the IFI44 related network composed of 76 nodes and 75 edges was extracted, including one miRNA, 18 TFs, and 56 drugs (Figure 8C).

**TABLE 1:** Top five important biomarkers in each machine learning method.

Rank	RF	SVM	ANN	LR	XGBoost
1	PIRC285	IFI44L	**IFI44**	**IFI44**	**IFI44**
2	IFI44L	EIF2AK2	EIF2AK2	PIRC285	IFIT3
3	**IFI44**	ZBP1	IFI44L	XAF1	EIF2AK2
4	XAF1	**IFI44**	PIRC285	ZBP1	IFI44L
5	IFIT3	PARP9	XAF1	EIF2AK2	XAF1

RF, Random forest; SVM, Support-vector machine; ANN, Artificial neural network; LR, Linear regression; XGBoost, eXtreme Gradient Boosting.

**FIGURE 9:**
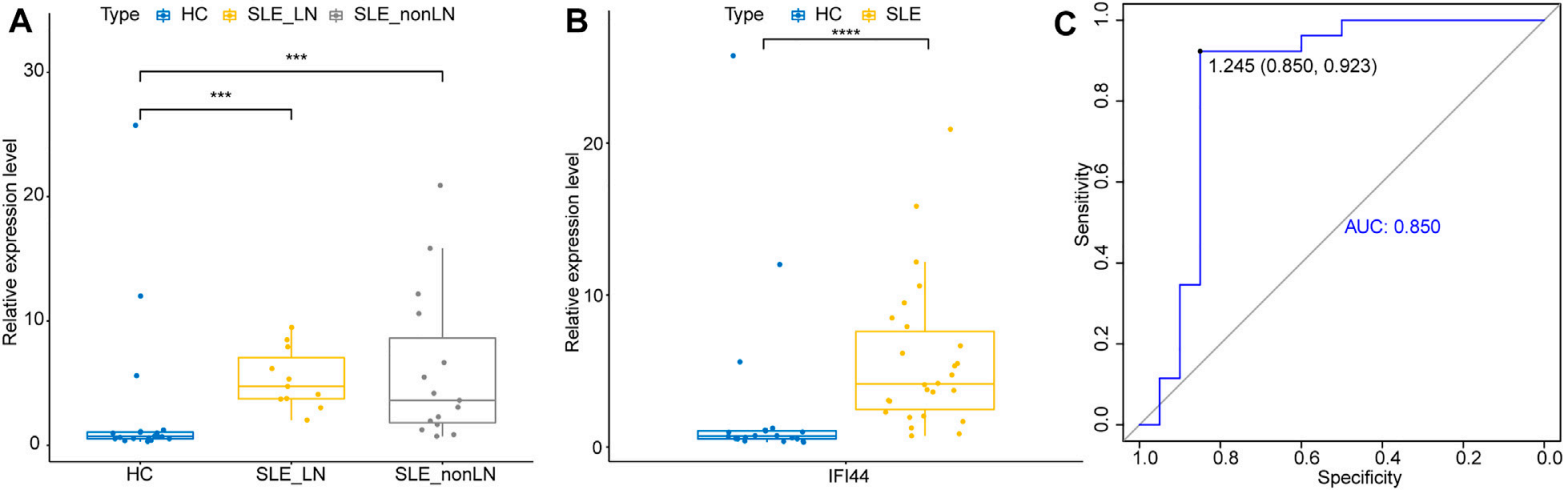
Validation of IFI44 as SLE diagnosis biomarker in an independent cohort. **(A)** Expression of IFI44 in SLE patients and healthy controls. **(B)** Expression of IFI44 in SLE patients with or without LN and healthy controls. **(C)** ROC curves of IFI44 for SLE diagnosis. ***p < 0.001, ****p < 0.0001.

To further explore whether IFI44 could be a reliable biomarker, the expression levels of IFI44 were identified by qRT-PCR in an independent cohort of 26 SLE patients and 20 healthy controls. The main clinical features of patients and controls are summarized in Table 2. Results showed that compared to that in healthy controls, the expression of IFI44 in SLE patients’ PBMCs was significantly higher no matter whether lupus nephritis (LN) was present (Figures 9A, B). ROC curve analysis showed that AUC for IFI44 was 0.850 when distinguishing SLE patients from healthy controls, and the diagnostic sensitivity and specificity were 0.923 and 0.850, respectively (Figure 9C).

**TABLE 2:** Clinical characteristics of SLE patients and healthy controls.

Clinical characteristics^#^	SLE (*n* = 26)	Healthy Control (*n* = 20)
Sex, male/female	2/24	2/18
Age (year)	36.77 ± 14.49	32.55 ± 9.99
Duration (year)	7.63 ± 6.96	
LN	11 (26)	
SLEDAI scores	11.5 ± 3.81	
ANA (Positive)	25 (26)	
Anti-ds-DNA antibody (Positive)	22 (26)	
Lupus anticoagulant (Positive)	10 (26)	
Leukocyte (10^9^/L)	5.43 ± 1.81	
Platelets (109/L)	179.8 ± 95.41	
CRP (mg/L)	9.95 ± 12.24	
ESR (mm/h)	19.2 ± 14.96	
C3 (g/L)	0.73 ± 0.46	
C4 (g/L)	0.1 ± 0.07	
IgG (g/L)	14.04 ± 8.02	
IgA (g/L)	2.65 ± 1.7	
IgM (g/L)	0.79 ± 0.64	
Serum creatinine (μmol/L)	70.81 ± 57.27	
24 h urine protein (Positive)	10 (11)	

^#^ LN, lupus nephritis; SLEDAI, systemic lupus erythematosus disease activity index; ANA, antinuclear antibody; Anti-dsDNA, antibody, anti-double stranded deoxyribonucleic acid antibody; CRP, C-reactive protein; ESR, erythrocyte sedimentation rate; C3/C4 complement 3/complement four; Igs, immunoglobulins.

## Discussion

In this study, two gene expression profile datasets were integrated and analyzed by multiple bioinformatic approaches. 11 DEGs between SLE and control samples were identified and analyzed by GO and KEGG. The results of GO analysis in the biological process have mainly enriched the response of type I and type II interferon (IFN) and the regulation of cytokines, while KEGG pathway analysis showed these DEGs were involved in the pathway of necroptosis. There was sufficient evidence to support that the impaired expression of type I IFN and its related genes were widely involved in the pathology of SLE (Postal et al., 2020), and the activity of type I IFN is related to the level of circulating type II IFN (Oke et al., 2019). The dysregulated secretion of cytokines and associated impairment of immune regulation is a key factor influencing the symptoms and disease activity in SLE patients (Howe and Leung, 2019). Necroptosis, a specialized programmed cell death, is a regulated mode of necrotizing cell death mediated by the RIP1 and RIP3 kinases, the hyperactivation of which leads to autoimmunity (O'Donnell et al., 2018). It has been reported that elevated IFN signaling in SLE increased necroptosis, leading to tissue damage (Sarhan et al., 2019). Further, necroptosis was also associated with B cell reduction in SLE patients (Fan et al., 2014).

Through PPI analysis, we found that IFI44, IFI44L, EIF2AK2, IFIT3, IFITM3, ZBP1, TRIM22, PRIC285, XAF1, and PARP9, interacted with each other and had strong positive correlations, and all of them have high diagnostic efficiency in different datasets. Therefore, these 10 genes could be used as diagnostic biomarkers for SLE. The expression of IFI44 is induced by interferon, which may reflect the whole blood interferon signature in SLE (Strauß et al., 2017). DNA methylation is often inversely proportional to the transcriptional activity of genes, and PARP9 and IFI44L showed marked hypomethylation in a variety of immune cells in SLE patients (Ulff-Møller et al., 2018), including CD4^+^ T cells, monocytes, granulocytes, and B cells. Hypomethylation of the IFI44L promoter region has excellent sensitivity and specificity for diagnosing SLE and distinguishing it from other autoimmune diseases (Zhao et al., 2016). EIF2AK2 is highly expressed in SLE and selectively modulates immune responses and transcription of SLE-related histone genes by targeting TFs (Ge et al., 2021). IFIT3 promotes the production of type I IFN and other pro-inflammatory cytokines in SLE patients by positively regulating the cGAS-STING signaling pathway, aggravating the symptoms of SLE (Wang et al., 2018). IFITM3 is an interferon-induced transmembrane protein whose role in SLE is unclear, but it can inhibit the production of IL-6 (Stacey et al., 2017) and regulate the differentiation of T helper cells (Yánez et al., 2020), so it may help to regulate the inflammatory response and immune regulation in SLE. ZBP1 is closely related to necroptosis. Stimulated by IFN, ZBP1 protein can interact with RIPK3 to initiate RIPK3-dependent necroptosis (Yang et al., 2020). TRIM22 is a viral restriction factor that may play a role in certain autoimmune diseases such as multiple sclerosis (Jefferies et al., 2011). PRIC285 is a transcriptional coactivator involved in PPAR-γ signaling (Fairfax et al., 2012), and PPAR-γ can inhibit the activation of macrophages and regulate their differentiation, improving SLE symptoms (Kiss et al., 2013). XAF1 can increase p53 transcriptional activity (Pinto et al., 2020), and p53-dependent apoptosis has been implicated in the pathogenesis and disease activity of SLE (Chen et al., 2021).

The organ damage caused by SLE is attributed to the deposition of immune complexes on the one hand and the infiltration of activated immune cells on the other hand (Apostolidis et al., 2011). Therefore, we compared the immune cell infiltration between SLE and control samples in GSE65391 and found that the proportions of 18 immune cells were significantly different between the two groups, of which 10 were significantly associated with the screened diagnostic genes. Among them, the proportion of central memory CD8+ T cells was significantly high, and there was a strong positive correlation with diagnostic genes, which may be associated with the maintenance of chronic inflammation (Liu et al., 2007). It is generally believed that the Th2-dominated Th1/Th2 imbalance and the pathogenesis of SLE are intimately connected (Jiang et al., 2021), and our results showed that the diagnostic biomarkers have a significant positive correlation with the increased frequency of Th2, but not with Th1, which supported the reliability of the diagnostic biomarkers screened by us.

In addition, we constructed the miRNA-diagnostic biomarker-TF network to explore the regulatory mechanisms of the selected genes. MicroRNA (miRNA) is one of the main epigenetic regulators of SLE-related genes. A considerable amount of research progress has been made in the development of biomarkers and therapeutic methods based on miRNA (Hong et al., 2020). The network indicated that hsa-miR-6799-5p and hsa-miR-6759-5p, two tumor suppressor-related miRNAs, could interact with EIF2AK2 and IFI44L simultaneously. Hsa-miR-6759-5p can regulate the PI3K/AKT pathway that plays an important role in chronic inflammation by targeting IGF2 (Liu et al., 2020), while the molecular function of hsa-miR-6799-5p is still unclear. We also used the CTD database to predict the drugs associated with the diagnostic genes and established a drug-gene network, which can provide a reference for constructing new treatment options or mining potential pathogenic factors for SLE. For example, the (+)-JQ1 compound simultaneously targeted seven diagnostic genes in the network, and *in vitro* treatment of CD4+ T cells from SLE patients with JQ1 has been reported to reverse immune dysregulation and reduce inflammatory cytokines such as IFN-γ and IL-21 (Gao et al., 2018), suggesting that it could be a potential SLE therapeutic drug. The notorious carcinogen Benzo(a)pyrene also affects multiple diagnostic genes, but it is also an immunomodulator that can act as a ligand for aryl hydrocarbon receptors to alleviate arthritis symptoms in certain autoimmune diseases such as RA (Hui and Dai, 2020).

In this study, the RobustRankAggreg algorithm was used to evaluate the expression consistency of diagnostic genes in multiple datasets, and multiple machine learning algorithms were used to evaluate the contribution of different diagnostic genes to distinguish disease and control samples, to make the identification of biomarkers more accurate. Finally, the results showed that IFI44 had the highest contribution, suggesting that it may be the optimal SLE diagnostic biomarker. IFI44 is a type I IFN signature gene, which was hypomethylated in SLE patients (Joseph et al., 2019) and negatively regulated the innate immune response induced by the virus (DeDiego et al., 2019). Therefore, it may be related to the immune imbalance of autoimmune diseases. IFI44 has been considered as a key diagnostic biomarker in various diseases, including Sjogren’s syndrome (Xu et al., 2021) and psoriasis (Wang et al., 2020). A recent study showed that IFI44 can serve as a key biomarker for LN from IgA nephritis and healthy controls, and was associated with LN disease activity (Shen et al., 2021), suggesting that IFI44 was not only involved in the damage of immune complexes to the kidney but also closely related to the pathogenesis of SLE. Lupus nephritis is a frequent and severe complication of SLE, occurs in about 40% of SLE patients, which often indicates a poor prognosis (Gasparotto et al., 2020). The subjects included in our study were not limited to lupus nephritis, so it can better reflect the value of IFI44 in the diagnosis of SLE. The qRT-PCR results showed the up-regulation of IFI44 differed significantly between SLE patients with or without LN and healthy controls, indicating that IFI44 might be a reliable SLE diagnostic biomarker.

However, our study has certain limitations. Firstly, the samples of one of the datasets we used, GSE65391, were mainly from pediatric patients, and it is difficult to determine whether the age factor affected the research results. Secondly, we need further experiments to verify our findings, such as validating in a larger scale and rigorous trial, evaluating the expression of the other biomarkers we identified, and comparing the expression of IFI44 in various autoimmune diseases.

## Conclusion

In conclusion, we found ten potential diagnostic biomarkers (IFI44, IFI44L, EIF2AK2, IFIT3, IFITM3, ZBP1, TRIM22, and PRIC285) for SLE by integrating bioinformatics methods, and discovered the potential of IFI44 as an optimal biomarker by five machine learning algorithms. The qRT-PCR and ROC curve analysis were performed to validate the diagnostic performance of IFI44 in an independent cohort. Immune cell infiltration showed the proportion of central memory CD8^+^ T cells was significantly high and positively correlated with selected biomarkers in SLE patients. The construction of miRNA-diagnostic biomarker-TF regulatory network and drug-gene network provides ideas for further exploring the pathogenesis at the genetic level and treatment of SLE.

## Data Availability

The datasets presented in this study can be found in online repositories. The names of the repository/repositories and accession number(s) can be found in the article/Supplementary Material.
