# Supplementary material for: Identification of Diagnostic Biomarkers in Systemic Lupus Erythematosus Based on Bioinformatics Analysis and Machine Learning
Source: Front Genet. 2022 Apr 14;13:865559. doi: 10.3389/fgene.2022.865559 (PMC9047905; doi:10.3389/fgene.2022.865559)
Supplement: Supplementary file 1 [file DataSheet1.ZIP › Supplementary_Material.docx]

Supplementary Material

# Supplementary Figures and Tables

## Supplementary Figures

**
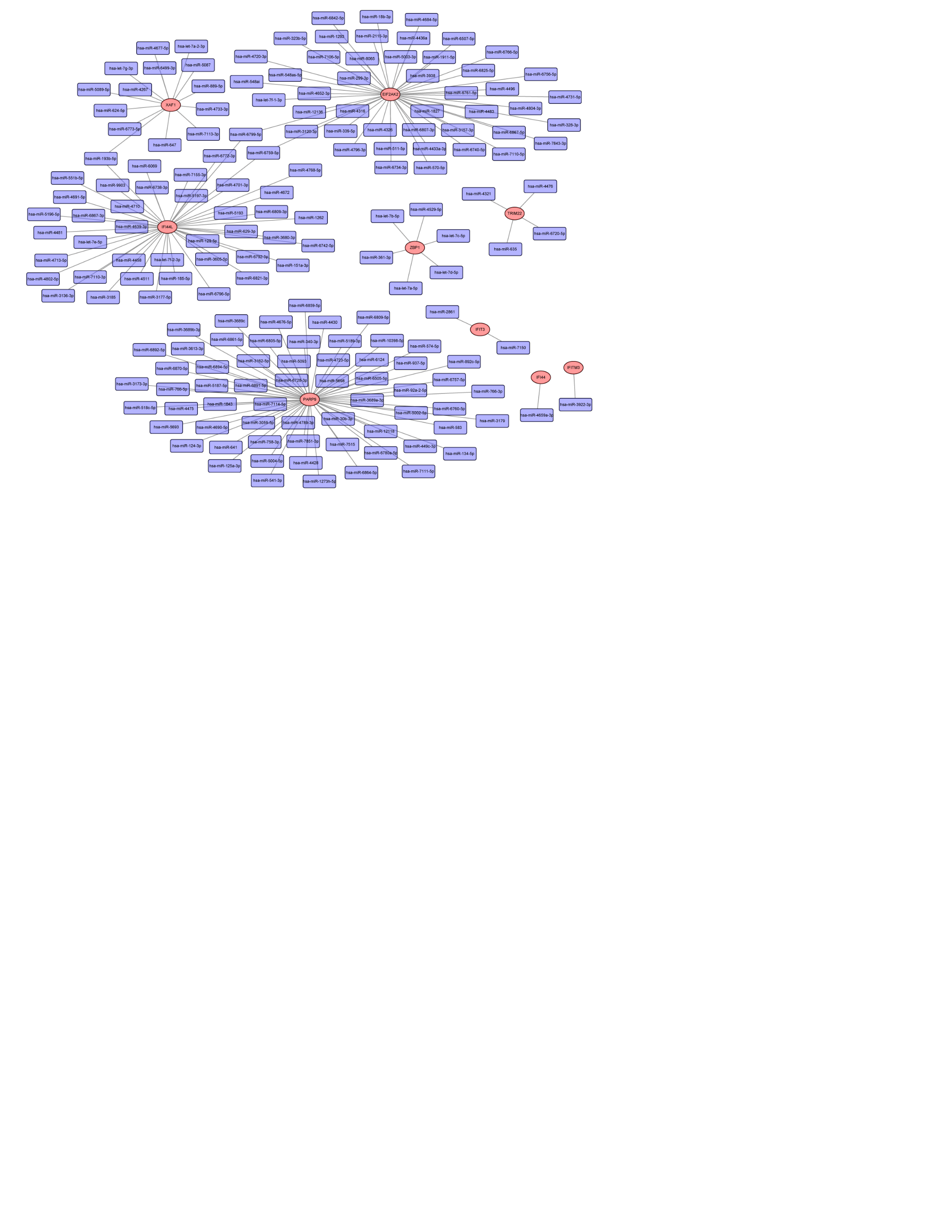
**

**Supplementary Figure 1.** Biomarker-miRNA regulatory network. The orange ellipses represent the biomarkers and the purple round rectangles represent the miRNAs.


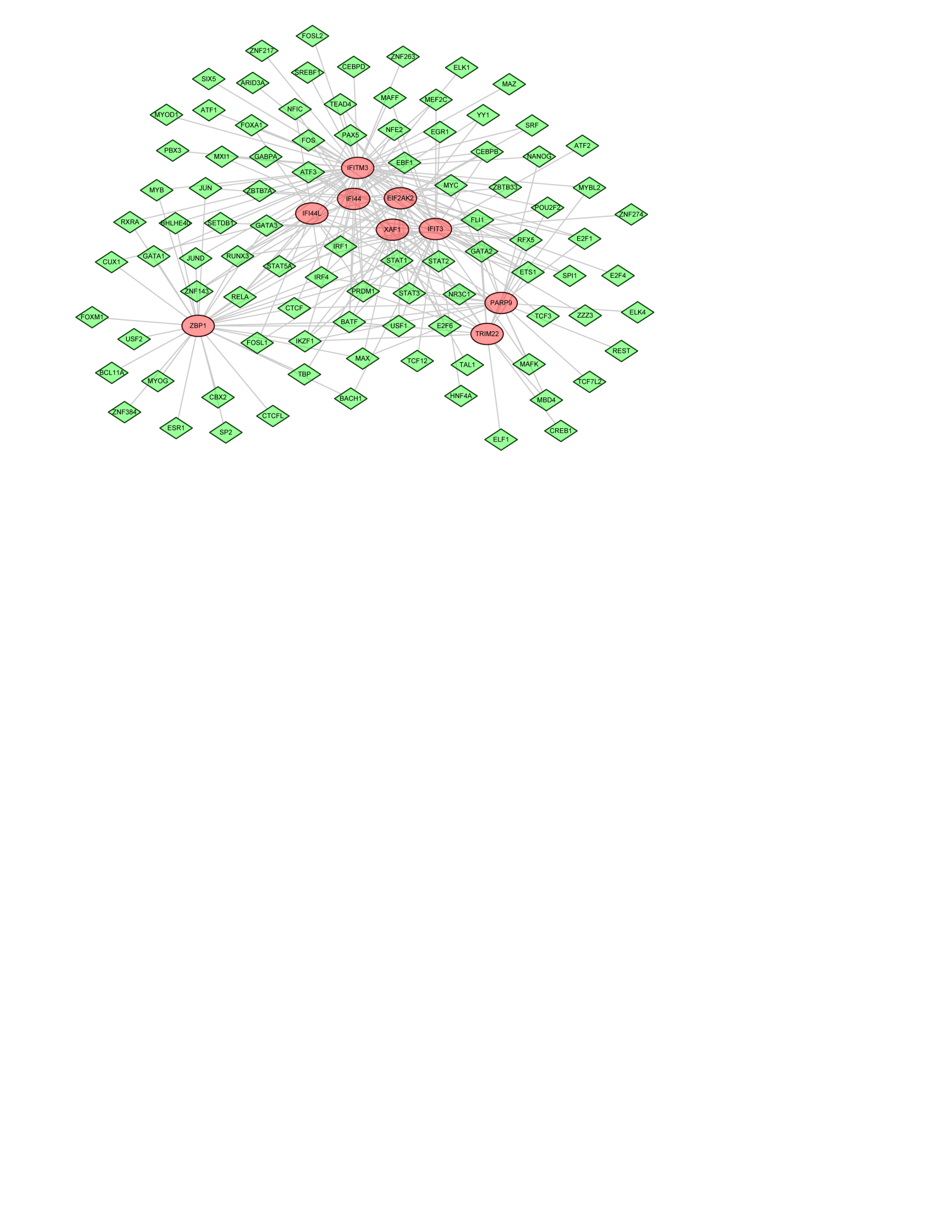


**Supplementary Figure2.** TF-biomarker regulatory network. The orange ellipses represent the biomarkers and the green diamonds represent the TFs.

## Supplementary Tables

**Supplementary Table 1.** Primers used for qRT-PCR

**Supplementary Table 2.** A total of 161 DEGs in GSE65391.

**Supplementary Table 3.** A total of 125 DEGs in GSE72509.

**Supplementary Table 4.** 11 accurate DEGs screened by RobustRankAggreg method.

**Supplementary Table 5.** The correlation network of DEGs.

**Supplementary Table 6.** The predicting drugs targeting biomarkers by CTD.

**Supplementary Table 7.** The result of ssGSEA algorithm for infiltration of 28 immune cells in each sample.
