# Supplementary material for: Identification of Diagnostic Biomarkers in Systemic Lupus Erythematosus Based on Bioinformatics Analysis and Machine Learning
Source: Front Genet. 2022 Apr 14;13:865559. doi: 10.3389/fgene.2022.865559 (PMC9047905; doi:10.3389/fgene.2022.865559)
Supplement: Supplementary file 1 [file DataSheet1.ZIP › Table S1.docx]

| Gene names | Primers（5’-3’） |
| --- | --- |
| β-actin-F | CATGTACGTTGCTATCCAGGC |
| β-actin-R | CTCCTTAATGTCACGCACGAT |
| IFI44-F | TCTTTCTGACATCTCGGTGGT |
| IFI44-R | CAGCCCATAGCATTCGTCT |
